# Insulin-like growth factor binding protein-1 and insulin in polycystic ovary syndrome: a systematic review and meta-analysis

**DOI:** 10.3389/fendo.2023.1279717

**Published:** 2023-12-15

**Authors:** Yuxin Jin, Fei Sun, Aili Yang, Xinwen Yu, Yi Li, Shengru Liang, Xiaorui Jing, Kai Wang, Lan Zhang, Sa Xiao, WenCheng Zhang, Xiaoguang Wang, Guohong Zhao, Bin Gao

**Affiliations:** ^1^ Department of Endocrinology, Tangdu Hospital, Air Force Medical University, Xi’an, Shaanxi, China; ^2^ Department of Gynaecology and Obstetrics, Tangdu Hospital, Air Force Medical University, Xi’an, Shaanxi, China

**Keywords:** insulin-like growth factor binding protein-1 (IGFBP-1), insulin-like growth factor-1 (IGF-1), hyperinsulinemia, insulin resistance (IR), hyperandrogenism, polycystic ovary syndrom (PCOS)

## Abstract

**Background:**

Insulin-like growth factor binding protein-1 (IGFBP-1) is considered a decline in polycystic ovary syndrome (PCOS), but it remains controversial that whether such reduction is attributed to obesity.

**Aims:**

This systematic review aims to explore whether IGFBP-1 is reduced in PCOS, and whether such reduction is associated with obesity.

**Results:**

Our pooled study included 12 studies with a total of 450 participants. IGFBP-1 levels in PCOS were significantly lower than that in non-PCOS (SMD (95%CI)=-0.49(-0.89, -0.09), *P*=0.02). No significant difference in IGFBP-1 levels between patients with or without PCOS classified by BMI. Whilst, stratification by PCOS status revealed a significant decrease in IGFBP-1 in overweight (SMD (95%CI)=-0.92(-1.46, -0.37), P=0.001). When comparing fasting insulin in the same way, PCOS patients had significantly elevated fasting insulin level but not statistically declined IGFBP-1 after classified by BMI.

**Conclusion:**

This meta-analysis provides evidence that the decrease of IGFBP-1 in PCOS was more strongly influenced by comorbid obesity than by PCOS itself. Additionally, contrast to previous findings that insulin significantly suppresses IGFBP-1, our results suggested that the suppression of PCOS-related hyperinsulinemia on IGFBP-1 seemed diminished. Overall, our work may provide a novel perspective on the mechanism between insulin and IGFBP-1 underlying PCOS development.

## 1 Introduction

Polycystic ovary syndrome (PCOS) is a prevalent endocrine disorder among reproductive-aged, who are at higher risk of developing metabolic disorders, infertility and birth complications (1). Hyperandrogenism and hyperinsulinemia have been demonstrated as the two predominant interrelated mechanisms involved in the development of PCOS. Insulin-like growth factor-1 (IGF-1) axis has been reported to play an important role in regulating the androgen and insulin resistance level (2–4). Specifically, hyperinsulinemia could suppress hepatic insulin-like growth factor binding protein-1 (IGFBP-1) gene transcription, reducing free IGFBP-1 in blood. Lower IGFBP-1 could increase free IGF-1 level by combining less IGF-1, who acts synergistically with LH to increase thecal androgen production (5, 6). And in turn hyperandrogenism levels may induce muscular changes leading to reduced insulin-mediated glucose uptake, ultimately resulting in insulin resistance (IR). Again, IR could aggravate hyperinsulinemia (7, 8). Thus, IGFBP-1 may play a critical role in the pathogenesis in the development of PCOS disease.

PCOS patients had lower levels of IGFBP-1 (9–11), which have been associated with early miscarriage and ovulation obstacle (12, 13). Generally, lower levels of IGFBP-1 may affect the progression of PCOS through obesity-related mechanisms (14). For example, some studies have found that this association only appears in obese individuals with PCOS (9, 10). However, there is still controversy surrounding this perspective. Several studies suggest a correlation between IGFBP-1 and PCOS regardless of obesity (15, 16). Therefore, it is necessary to explore the role of IGFBP-1 in PCOS to better understand its development. Moreover, considering that excessive androgen can lead to a decrease in IGFBP-1 levels independently of obesity, we would like to explore whether IGFBP-1 is correlated with PCOS itself even after adjusting for obesity.

This meta-analysis aims to pool eligible observational studies so far to investigate whether level of IGFBP-1 is predominantly correlated with obese PCOS patient and the possible underlying mechanism in it.

## 2 Materials and methods

### 2.1 Search strategy

In this meta-analysis, we followed the Preferred Reporting Items for Systematic Reviews and Meta-Analyses (PRISMA) guidelines. A comprehensive search was conducted using six electronic databases, namely PubMed, EMBASE, Cochrane Library, Web of Science, Ovid MEDLINE, and Scopus, up to February 2023. The search strategy information is available in the **Supplementary files** (Search strategy). We are applying for the registration in PROSPERO with ID: CRD42023428432.

### 2.2 Study selection

The inclusion criteria were as follows: (1) studies that measured IGFBP-1 and insulin levels in PCOS and non-PCOS women; (2) studies that compared IGFBP-1 and insulin levels among different weight women with or without PCOS; or (3) studies written in English. Diagnostic criteria for PCOS are shown in **Table 1**.

**Table 1:** Study characteristics of the included studies.

Study, year		PCOS Definition	Obesity Definition	Characteristics of Participants (Country)	Assay of IGFBP-1	Fasting	Mean age, years			Mean BMI, kg/m2
Firmansyah, A et al. (2018) (11)		Rotterdam 2003 criteria 2 out of 3 criteria: 1. Oligo-and/or anovulation, 2. Clinical and/or biochemical signs of hyperandrogenism, 3. Polycystic ovaries	BMI > 25 kg/m^2^	Total 60 adults PCOS (Indonesia): (1)43 overweight+PCOS (2)17 non-overweight+ PCOS	ELISA	Yes	Overweight PCOS: 29.5 ± 3.98 Non-overweight PCOS: 28.4 ± 2.12			NA
Silfen et al. (2003) (10)		1. Hyperandrogenism (1) free testosterone > 6.3 pg/ml (2) testosterone >55 ng/dl (3) Δ4-androstenedione > 245 ng/dl, DHEAS > 248 g/dl 2. Oligomenorrhea/amenorrhea	BMI > 25 kg/m^2^	Total 48 adolescents (USA): (1)11 non-obese+PCOS (2)22 obese + PCOS (3)15 obese + non-PCOS	ICMA	Yes	Nonobese PCOS 16.1 ± 1.9 Obese PCOS 15.5 ± 1.4 Obese non-PCOS 14.4 ± 1.5			Nonobese PCOS 22.5 ± 1.5 Obese PCOS 35.9 ± 6.2 Obese non-PCOS 35.8 ± 7.1
Garcia-Rudaz et al. (2002) (16)		1. Hyperandrogenism (hirsutism, evaluated by a Ferriman–Gallwey score of at least 9 and/or acne) 2. Oligomenorrhea or amenorrhea, 3. And elevated serum levels of androstenedione and/or testosterone.	BMI >25 kg/m^2^	Total 23 adolescents (USA): (1)13 PCOS (2)10 non-PCOS	IRMA	Assumed	PCOS 16.4 ± 0.6 Non-PCOS: 16.5 ± 0.5			PCOS 24.3 ± 1.6 Non-PCOS: 22.3 ± 1.0
Kowalska, I et al. (2001) (17)		1. Clinical findings (oligo/amenorrhea and hirsutism); 2. Laboratory data (testosterone elevated or in the upper limit of normal) 4. All patients had polycystic ovaries (ultrasonography).	BMI > 27.5 kg/m^2^	Total 53 adolescents (Poland): (1)23 obese+ PCOS (2)19 obese+ non-PCOS (3)11 nonobese+ non-PCOS	RIA	Yes	Obese+ PCOS 25.3 ± 4.8 Obese+ non-PCOS 27.9 ± 7.3 Nonobese+ Non-PCOS 30.4 ± 5.7			Obese+ PCOS 34.7 ± 6.0 Obese+ non-PCOS 36.2 ± 6.0 Nonobese+ Non-PCOS 21.9 ± 2.0
Carmina, E et al. (1997) (9)		1. Hyperandrogenism 2. Chronic anovulation 3. Ultrasound diagnosis of PCOS: (1) both ovaries of ≥10 peripherallyoriented cysts (<8 mm) (2) increased ovarian volume (3) increased stromal density	NA	Total 55 adults (USA): (1)15 ovulation+ polycystic ovaries (PAO) (2)15 ovulation+ normal-ovaries (NAO) (2)25 PCOS	IRMA	Yes	PAO 28.0 ± 1.0 NAO 28.0 ± 1.0 PCOS 25.0 ± 1.0			PAO 21.1 ± 0.6 NAO 20.7 ± 0.3 PCOS 25.24 ± 2.7
Morales, A J et al. (1996) (18)		1. Oligomenorrhe 2. Levated androstenedione and/or testosterone 3. Increased LH/FSH ratio (>2.0) 4. Ultrasound evidence of polycystic ovaries	Obsity: BMI >30 kg/m^2^; Lean: BMI <23 kg/m^2^	Total 32 adults (USA): (1)8 obese+ PCOS (OPCOS) (2)8 obese+ non-PCOS (OC) (3)8 lean + PCOS (LPCOS) (4)8 lean + non-PCOS (LC)	IFMA	Yes	OPCOS 27.9 ± 2.1 OC 26.7 ± 1.9 LPCOS 26.7 ± 1.9 LC 28.2 ± 1.3			OPCOS 36.1 ± 2.4 OC 32.9 ± 1.1 LPCOS 21.4 ± 0.7 LC 21.4 ± 0.4
Morris, D V et al. (1996) (19)		1. Amenorrhea/oligomenorrhea 2. Hirsutism (grade 2 or higher, on the face and abdomen) 3. Levated testosterone and androstenedione	BMI >25 kg/m^2^	Total 25 adults (Canada): (1)15 PCOS (2)10 non-PCOS	RIA	Yes	PCOS 27.0 ± 0.3 Non-PCOS 29.0 ± 0.6			PCOS 26.6 ± 1.59 Non-PCOS 24.0 ± 0.9
Buyalos R. P. et al. (1995) (20)		1. Perimenarcheal onset of oligomenorrhe or amenorrhea 2. At least facial hirsutism. 3. Ultrasonographic evaluation of ovarian morphologic 4. Absence of virilized	BMI >25 kg/m^2^	Total 36 adults (USA) (1)9 obese + PCOS (2)10 obese + non-PCOS (3)7 nonobese + PCOS (4)10 nonobese + non-PCOS	IRMA	Yes	Obese+PCOS 31.0 ± 2.0 Obese+non-PCOS 29.0 ± 2.0 Nonobese+PCOS 29.0 ± 1.0 Nonobese+non-PCOS 27.0 ± 1.0			Obese+PCOS 35.9 ± 1.7 Obese+non-PCOS 33.7 ± 2.5 Nonobese+PCOS 21.8 ± 0.8 Nonobese+non-PCOS 21.6 ± 0.5
Insler et al. (1993) (21)		1. Sonographic appearance of polcystic ovaries 2. Clinical and hormonal criteria, including: (1) oligomenorrhoea (2) or elevated basal serum LH concentrations (3) or hirsutism or acne.	BMI >25 kg/m^2^	Total 8 PCOS(Israel) (1)4 obese+PCOS (2)4 nonobese+ PCOS	IFMA	Assumed	Obese+PCOS 27.3 ± 2.9 Nonobese+PCOS 25.5 ± 2.9			Obese+PCOS 34.7 ± 2.3 Nonobese+PCOS 20.5 ± 0.8
Tiitinen, A E et al. (1993) (22)		Clinicla symtomes, laboratory findings and typical appearance of the ocaries in ultrasonography, including: (1) hirstute, amenorrhaea, irregular anovulatory cycles (2) elevated androstenedione and/or testosterone	Obese BMI >25 kg/m^2^ Lean BMI ≤25 kg/m^2^	Total 28 adults(Finland) (1)10 obese+PCOS (2)13 lean+PCOS (3)5 lean+non-PCOS	RIA	Yes	Obese+PCOS 25.7 ± 0.8 Lean+PCOS 28.6 ± 0.8 Lean+non-PCOS 25.6 ± 1.2			Obese+PCOS 32.5 ± 1.4 Lean+PCOS 23 ± 2.2 Lean+non-PCOS 22.7 ± 1.0
Laatikainen et al. (1990) (23)		Hirsute or oligomenorrhea	NA	Total 26 adults(Finland) (1)7 obese+PCOS (2)13 nonobese+ PCOS (3)6 nonobese+ non-PCOS	RIA	Yes	Obese+PCOS 29.7 ± 2.6 Nonobese+PCOS 24.2 ± 1.8 Nonobese+non-PCOS 28.0 ± 2.4			Obese+PCOS 32.3 ± 1.64 Nonobese+PCOS 20.9 ± 2.5 Nonobese+non-PCOS 21.5 ± 0.56
Iwashita M et al. (1990) (24)		1. Menstrual disturbances 2. Polycystic ovary by ultrasound 3. Hyperandrogenemia 4. LH/FSH > 2.5	BMI < 24 kg/m^2^	Total 56 adults(Japan) (1)26 nonobese+PCOS (2)30 nonobese+ nonPCOS	ELISA	Yes	NA			NA	

BMI, body mass index; DHEAS, dehydroepiandrosteronesulfate; ELISA, enzyme-linked immunosorbent assay; IGFBP-1, insulin-like growth factor-binding protein-1; ICMA, immunochemiluminometric assay; IRMA, immunoradiometric assay; IFMA, immunofluorometric assay; PCOS, polycystic ovary syndrome; RIA, radioimmunoassay; SHBG, sex hormone-binding globulin; NA, not available.

We excluded studies that (1) subjects who were drug-induced hirsutism, postmenopausal, pregnant, lactating, diabetes mellitus, hypertension, pituitary, adrenal or thyroid diseases (e.g., hyperprolactinemia, adrenal congenital hyperplasia, or hyperthyroidism), (2) subjects who were on medication or hormonal therapy or weight loss program before study to affect metabolism or menses, (3) subjects who were *in vitro* fertilization program or on pregnancy, (4) reanalyzed published data and review articles without original data, or (5) were case report.

### 2.3 Data extraction

The collected information encompassed: (1) basic demographic characteristics and (2) outcomes, such as fasting IGFBP-1, fasting insulin and HOMA-IR. In case of missing data, attempts were made to contact the authors concerned. GetData Graph Digitizer 2.26 was used to acquire data from figures if the original data were not available through connection.

### 2.4 Assessment of risk of bias

As our analysis included data from studies that were collected at a single point in time without intervention, we used the *Joanna Briggs Institute (JBI) Critical Appraisal Checklist for an analytical cross-sectional study* to assess the methodological quality.

The processes of study search, selection, data extraction and assessment of risk of bias were conducted independently by two authors (Yuxin Jin and Fei Sun). Any discrepancies were resolved by discussions among all authors.

### 2.5 Methods of analysis

STATA V.16 and Review Manager 5.4 were used to combine the data. Due to methodological heterogeneity in outcome assessment (14), the data were pooled and presented as the standardized mean difference (SMD) with 95% confidence interval (CI). Standard deviations (SD) were missing instead of the standard error (SE) calculated in some studies (9, 16, 18–24), in which SD is estimated from SE by $SE=SD/\sqrt{n}$ .

In case of *I* ^2^ values of >50% and *p* values of < 0.1, heterogeneity was considered significant, and a random-effect model, sensitivity analyses and subgroup meta-analyses were performed. Otherwise, fixed-effect model was conducted. The results were regarded as statistically significant when the *p* value was < 0.05. Funnel plots cannot reveal sufficient information on publication bias when the number of included studies in each meta-analysis was less than 10; therefore, Egger’s and Begg’s tests were used to assess publication bias.

## 3 Results

### 3.1 Data selection and study characteristics

**Figure 1:**
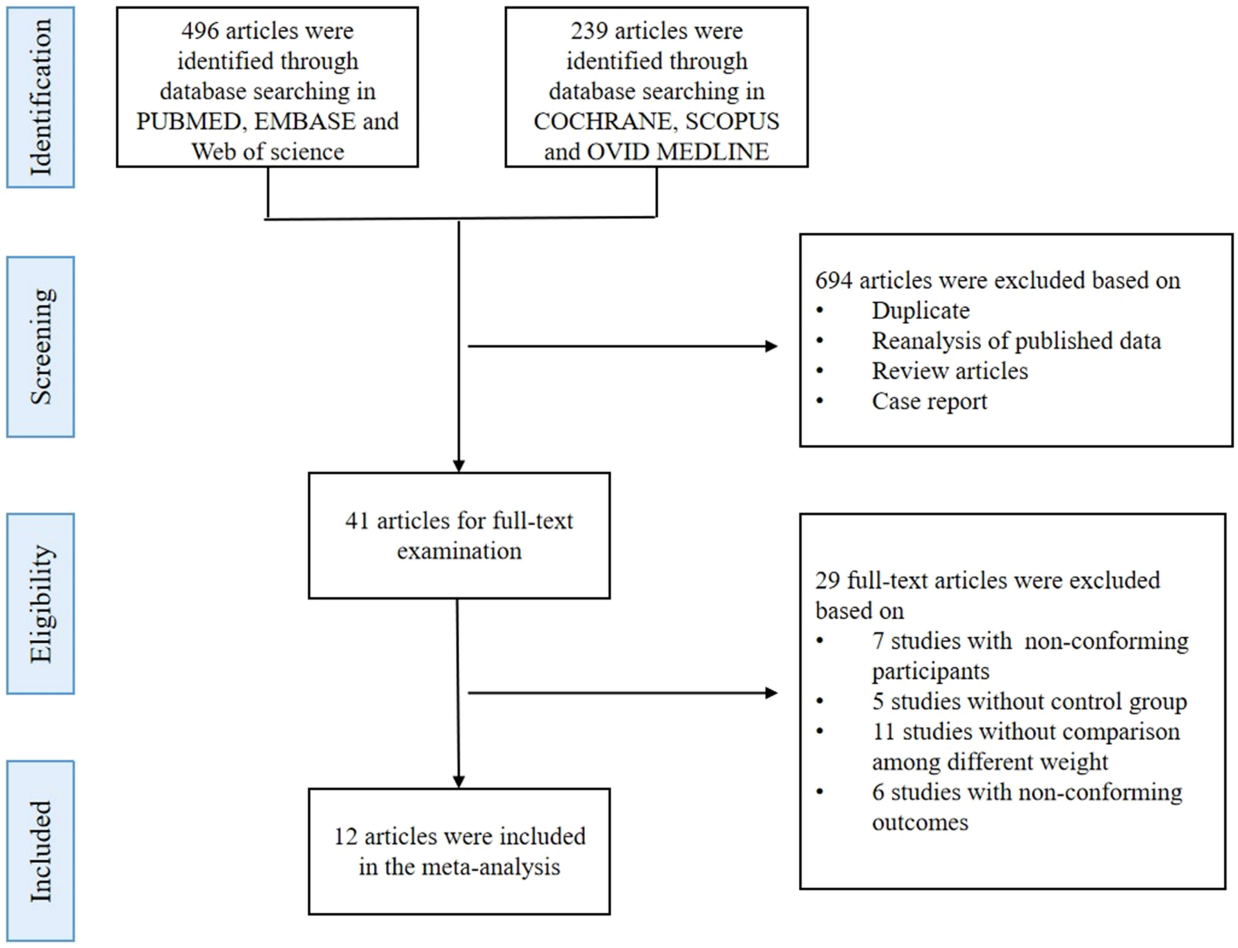
Flow diagram for literature searching.

A total of 735 studies were identified from the electronic databases. Upon screening the titles and abstracts, 694 records were excluded based on the exclusion criteria, leaving 41 full-text articles for further review. Finally, 12 studies were included in the quantitative synthesis based on inclusion and exclusion criteria (**Figure 1**) to compare IGFBP-1 in two populations: either PCOS versus non-PCOS, or an overweight subgroup versus the normal weight subgroup in either population. Thus, these 12 studies didn’t simultaneously enroll all comparison contents, which meant that every meta-analysis in our study could only enroll some of the 12 articles. The studies included a total of 450 participants, with an average age of 25.50 ± 5.63 years and an average BMI of 27.70 ± 7 kg/m^2^. All studies were observational, and their summary characteristics are shown in **Table 1**.

### 3.2 Quality assessment

It turns out that the quality was strong for most of those observational studies except 2003Silfen et al., 2002Garcia-Rudaz et al., 1997Carmina, E et al., which only underwent univariate analysis without clearly identifying confounding factors. Estimation details are shown in the **Supplementary File** (**Supplementary Data**, **Table S1**).

### 3.3 Primary analysis

#### 3.3.1 IGFBP-1 in participants of different weights with or without PCOS

##### 3.3.1.1 Meta-analysis for IGFBP-1 in PCOS patients versus the control population

**Figure 2:**
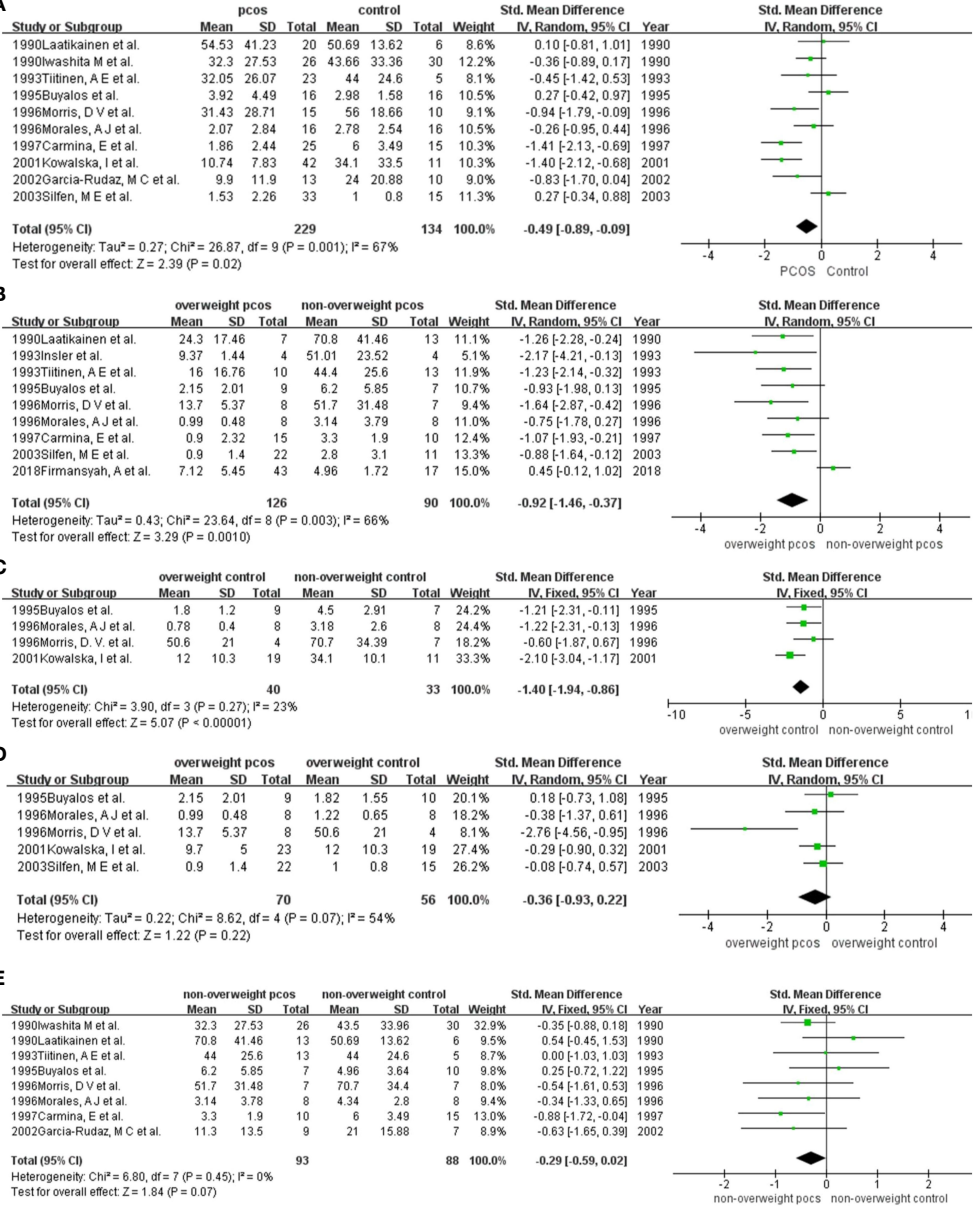
Comparison of IGFBP-1 in participants among different weights with or without PCOS: **(A)** PCOS versus the non-PCOS; **(B)** overweight PCOS versus non-overweight PCOS; **(C)** overweight non-PCOS versus non-overweight non-PCOS; **(D)** overweight PCOS versus overweight non-PCOS; **(E)** non-overweight PCOS versus non-overweight non-PCOS. PCOS, polycystic ovary syndrome; SD, standard deviation; 95%CI, confidence interval.

PCOS patients versus the control population data from 10 studies were pooled, and summary-level meta-analysis was performed (9, 10, 16–20, 22–24) (**Figure 2A**). Specifically, the mean (SD) IGFBP-1 levels by group were as follows: 1.53 (2.26) ng/ml to 54.53 (41.23) ng/ml for women with PCOS and 1.00 (0.8) ng/ml to 50.69 (13.62) ng/ml for women without PCOS. The aggregate data meta-analysis showed a significantly lower concentration of IGFBP-1 in women with PCOS compared to controls (SMD (95% CI) = -0.49 (-0.89, -0.09), p = 0.02) with *I* ^2^ = 67%. Furthermore, we used random-effects models to account for heterogeneity between studies, and the sensitivity analysis is shown in the **Supplementary Data**, **Figure S1**. Note that after the exclusion of three studies (9, 10, 17), the heterogeneity decreased (*I* ^2^ was reduced to 16%) with the significance of pooled results remaining unchanged (**Supplementary Data**, **Figure S2**). Funnel plots and Begg’s test and Egger’s test showed no evidence of publication bias (**Supplementary Data**, **Figure S3**, **S4**).

##### 3.3.1.2 Meta-analysis for IGFBP-1 in PCOS patients with or without overweight

We included a total of 9 studies in the quantitative synthesis (9–11, 18–22, 24) (**Figure 2B**). Specifically, the mean (SD) IGFBP-1 levels by group were as follows: 0.9 (1.4) ng/ml to 24.3 (17.46) ng/ml for overweight PCOS patients and 2.8 (3.1) ng/ml to 70.8 (41.46) ng/ml for the control group. The levels of IGFBP-1 were significantly lower in overweight PCOS patients than in nonoverweight PCOS patients, except for the study of Firmansyah, A et al. in 2018. The aggregate data meta-analysis showed a significantly lower concentration of IGFBP-1 in overweight PCOS patients than in nonoverweight PCOS patients (SMD (95% CI) = -0.92 (-1.46, -0.37), p = 0.001), with *I* ^2^ = 66% (**Figure 2B**). heterogeneity (*I* ^2^ = 66%). Sensitivity analysis suggested that the work of 2018Firmansyah, A et al. might explain the source of heterogeneity. Then, excluding the work of 2018Firmansyah, A et al., the heterogeneity disappeared as *I* ^2^ dropped to 0. Meanwhile, the difference in IGFBP-1 levels between the two groups remained significant. (**Supplementary Data**, **Figure S5**, **S6**).

Publication bias was identified according to the asymmetric funnel plot and Begg’s and Egger’s tests, with *p* values close to 0.05 (**Supplementary Data**, **Figure S7**, **S8**). Publication bias was identified according to the asymmetric funnel plot and Begg’s and Egger’s tests, with *p* values close to 0.05 (**Supplementary Data**, **Figure S7**, **S8**). In addition, publication bias disappeared after the study by Firmansyah, A et al. was removed. (**Supplementary Data**, **Figure S9**, **S10**).

##### 3.3.1.3 Meta-analysis for IGFBP-1 in the non-PCOS group with or without overweight

A total of 4 studies were included in the pooled analysis (17–20) (**Figure 2C**). Specifically, the mean (SD) IGFBP-1 levels by group were as follows: 0.78 (0.4) ng/ml to 50.6 (21) ng/ml for overweight women and 2.8 (3.1) ng/ml to 70.8 (41.46) ng/ml for nonoverweight women. The aggregate data meta-analysis showed a significantly lower concentration of IGFBP-1 in overweight women than in nonoverweight women (SMD (95% CI) = -0.14 (-1.94, -0.86), p < 0.001), with no heterogeneity. Although publication bias was observed (**Supplementary Data**, **Figure S11**), the association persisted after correction using the trim-and-fill method (**Supplementary Data**, **Figure S12**).

##### 3.3.1.4 Meta-analysis for IGFBP-1 in overweight participants with or without PCOS

Five studies including overweight participants were pooled (10, 17–20) (**Figure 2D**). Specifically, the mean (SD) IGFBP-1 levels by group were as follows: 0.9 (1.4) ng/ml to 13.7 (5.37) ng/ml for overweight PCOS women and 1 (0.8) ng/ml to 50.6 (21) ng/ml for overweight non-PCOS women. The meta-analysis showed no significant difference in IGFBP-1 levels between overweight PCOS women and overweight non-PCOS women (SMD (95% CI) = -0.36 (-0.93, 0.22), *p =* 0.22) with *I* ^2^ = 54% (**Figure 2D**). A sensitivity analysis supported the stability of the results after excluding 1996 Morris, D V et al., which led to the disappearance of heterogeneity (*I* ^2 =^ 0) (**Supplementary Data**, **Figure S13**, **S14**). No publication bias was found by Begg’s test or Egger’s test (**Supplementary Data**, **Figure S15**, **S16**).

##### 3.3.1.5 Meta-analysis for IGFBP-1 in nonoverweight participants with or without PCOS

A total of 8 studies were included in this meta-analysis (9, 16, 18–20, 22–24) (**Figure 2E**). Specifically, the mean (SD) IGFBP-1 levels by group were as follows: 3.3 (1.9) ng/ml to 70.8 (41.46) ng/ml for nonoverweight women with PCOS and 4.34 (2.8) ng/ml to 70.7 (34.4) ng/ml for nonoverweight women. Five studies (9, 16, 18, 19, 24) showed lower IGFBP-1 levels in PCOS women than in non-PCOS women. Two studies (20, 23) showed the opposite result, and the study of 1995Buyalos et al. showed no difference between the two groups. The meta-analysis revealed no significant difference in IGFBP-1 levels between PCOS and non-PCOS in nonoverweight people (SMD (95% CI) = -0.29 (-0.59, 0.02), *p =* 0.07). The statistical heterogeneity was negligible, *I* ^2^ = 0%. There was no indication of publication bias (**Supplementary Data**, **Figure S17**, **S18**).

#### 3.3.2 Fasting insulin in participants of different weights with or without PCOS

##### 3.3.2.1 Meta-analysis for fasting insulin in PCOS patients versus the control population

**Figure 3:**
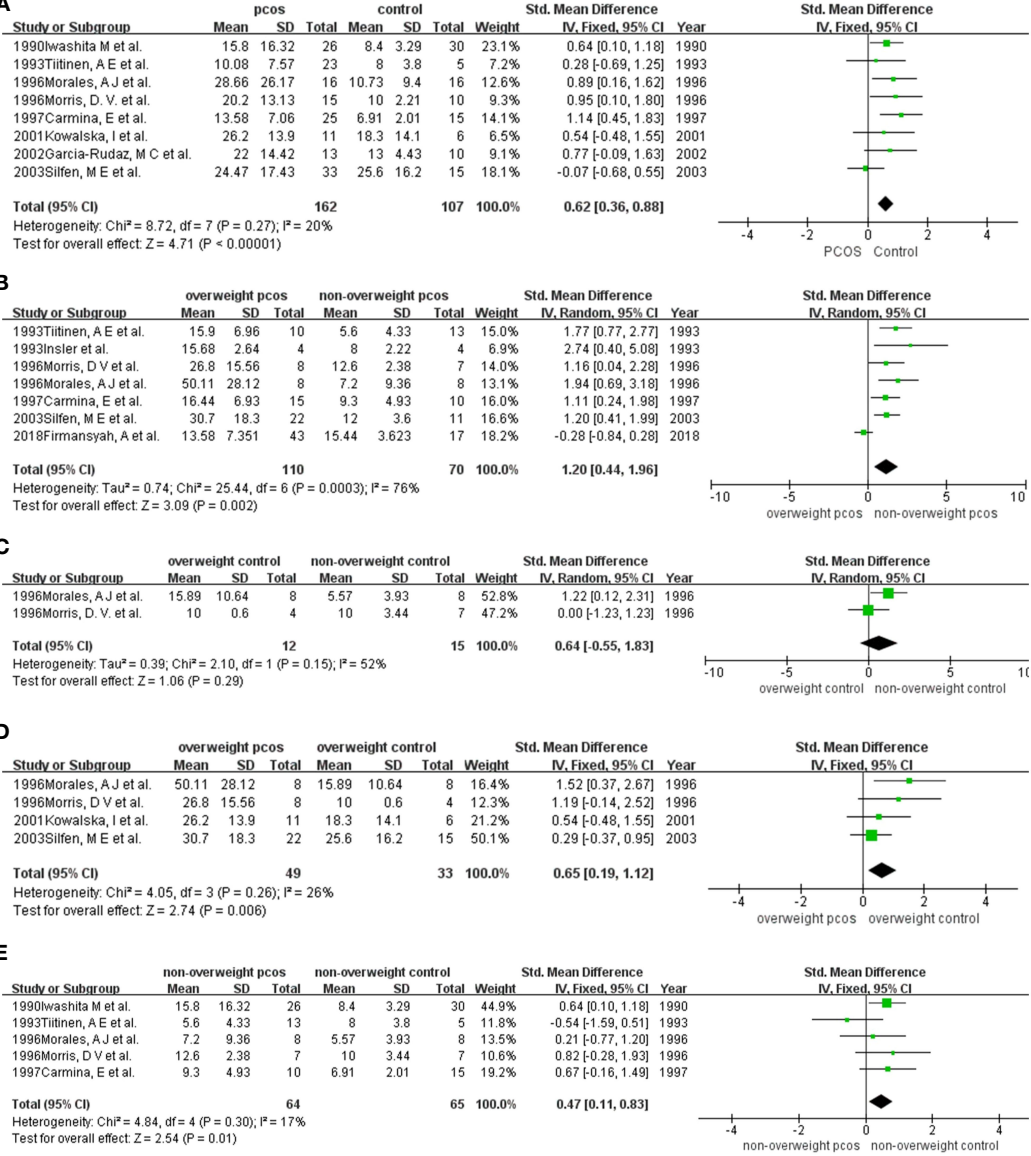
Comparison of fasting insulin in participants among different weights with or without PCOS: **(A)** PCOS versus the non-PCOS; **(B)** overweight PCOS versus non-overweight PCOS; **(C)** overweight non-PCOS versus non-overweight non-PCOS; **(D)** overweight PCOS versus overweight non-PCOS; **(E)** non-overweight PCOS versus non-overweight non-PCOS. PCOS, polycystic ovary syndrome; SD, standard deviation; 95%CI, confidence interval.

A total of 8 studies were included in the analysis (9, 10, 16–19, 22, 24) (**Figure 3A**). Specifically, the mean (SD) insulin levels by group were as follows: 13.58 (7.06) µIU/ml to 28.66 (26.17) µIU/ml for women with PCOS and 8.00 (3.8) µIU/ml to 25.6 (16.2) µIU/ml for women without PCOS. The pooled analysis revealed a significant increase in fasting insulin levels for women with PCOS compared with women without PCOS (SMD (95% CI) = 0.62 (0.36, 0.88), *p* < 0.001) with *I* ^2^ = 20%. No heterogeneity or publication bias was observed (**Supplementary Data**, **Figure S19**).

##### 3.3.2.2 Meta-analysis for fasting insulin in PCOS patients with or without overweight

Seven studies were included in this quantitative synthesis (9–11, 18, 19, 21, 22) (**Figure 3B**). Specifically, the mean (SD) insulin levels by group were as follows: 13.58 (7.35) µIU/ml to 50.11 (28.12) µIU/ml for overweight women and 5.6 (4.33) µIU/ml to 15.44 (3.62) µIU/ml for nonoverweight women. The levels of fasting insulin were significantly higher in overweight PCOS patients than in nonoverweight PCOS patients, except for the study of Firmansyah, A et al. in 2018. Higher fasting insulin levels were demonstrated in overweight PCOS by the meta-analysis (SMD (95% CI) = 1.20 (0.44, 1.96), *p =* 0.002), with statistical heterogeneity (*I* ^2^ = 76%) (**Figure 3B**). After excluding the study of 2018Firmansyah, A et al., the *I* ^2^ value was obviously reduced (*I* **^2^** = 0%) (**Supplementary Data**, **Figure S20**, **S21**). No publication bias was found based on Begg’s test and Egger’s test (**Supplementary Data**, **Figure S22**).

##### 3.3.2.3 Meta-analysis for fasting insulin in the non-PCOS group with or without overweight

Only 2 studies were included in the meta-analysis (18, 19) (**Figure 3C**). The results showed a higher level of fasting insulin in overweight women than in nonoverweight women, but the results were not significant (SMD (95% CI) = 0.64 (-0.55, 1.83), *p =* 0.29). Finally, due to the small number of studies included, we were unable to evaluate publication bias.

##### 3.3.2.4 Meta-analysis for fasting insulin in overweight participants with or without PCOS

Four studies were included in the pooled analysis (10, 17–19) (**Figure 3D**). Specifically, the mean (SD) fasting insulin levels by group were as follows: 26.2 (13.9) µIU/ml to 50.11 (28.12) µIU/ml for overweight women with PCOS and 10 (0.6) µIU/ml to 25.6 (16.2) µIU/ml for overweight women without PCOS. All studies reported significantly higher fasting insulin levels in women with PCOS than in those without PCOS. The meta-analysis supported that in the overweight population, PCOS patients had significantly increased fasting insulin levels compared with those without PCOS (SMD (95% CI) = 0.65 (0.19, 1.12), *p =* 0.006) (**Figure 2C**). There was no heterogeneity or publication bias observed in this pooled study (**Supplementary Data**, **Figure S23**).

##### 3.3.2.5 Meta-analysis for fasting insulin in nonoverweight participants with or without PCOS

Five studies were included in the analysis to compare fasting insulin levels between nonoverweight women with PCOS and those without PCOS (9, 18, 19, 22, 24) (**Figure 3E**). Specifically, the mean (SD) fasting insulin levels by group were as follows: 5.6 (4.33) µIU/ml to 15.8 (16.32) µIU/ml for nonoverweight women with PCOS and 5.57 (3.93) µIU/ml to 10 (3.44) µIU/ml for nonoverweight women. Five studies (9, 16, 18, 19, 24) showed lower IGFBP-1 levels in PCOS women than in non-PCOS women. Except for the study by 1993 Tiitinen, A E et al., other studies showed that fasting insulin levels in PCOS women were higher than those in nonoverweight women without PCOS. Our meta-analysis supported this finding statistically (SMD (95% CI) = 0.47 (0.11, 0.83), *p =* 0.01). There was no heterogeneity or publication bias observed in this pooled study (**Supplementary Figure S24**).

#### 3.3.3 HOMA-IR in participants among different weights with or without PCOS

##### 3.3.3.1 Meta-analysis for HOMA-IR in PCOS patients with or without overweight

**Figure 4:**
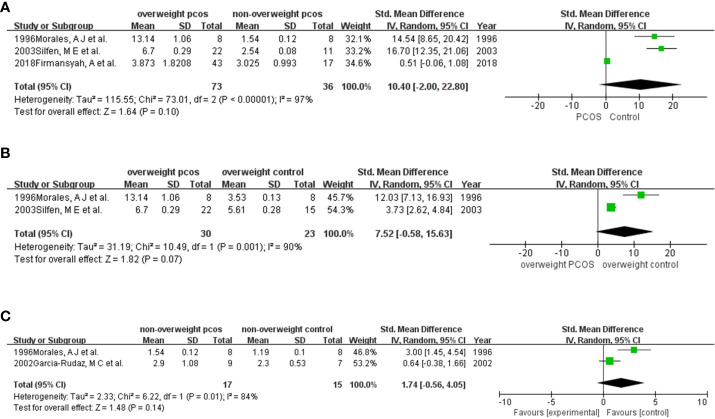
Comparison of HOMA-IR in participants among different weights with or without PCOS: **(A)** overweight PCOS versus non-overweight PCOS; **(B)** overweight PCOS versus overweight non-PCOS; **(C)** non-overweight PCOS versus non-overweight non-PCOS.

Three studies (10, 11, 18, 19) (**Figure 4A**) were enrolled in the meta-analysis. The results revealed stronger insulin resistance in overweight PCOS patients than in nonoverweight PCOS patients, although the results were not significant (SMD (95% CI) = 10.40 (-2.00, 22.80), *p =* 0.10).

##### 3.3.3.2 Meta-analysis for HOMA-IR in overweight participants with or without PCOS

There were only 2 studies (10, 18) (**Figure 4B**) pooled in this analysis. The results showed a higher HOMA-IR in women with PCOS than in those without PCOS (SMD (95% CI) = 7.52 (-0.58, 15.63), *p =* 0.07).

##### 3.3.3.3 Meta-analysis for HOMA-IR in nonoverweight participants with or without PCOS

Two studies (16, 18) (**Figure 4C**) were included in this meta-analysis. The results supported that HOMA-IR was higher in PCOS than in non-PCOS women who were nonoverweight. (SMD (95% CI) = 1.74 (-0.56, 4.05), *p =* 0.14).

##### 3.3.3.4 Comparison of HOMA-IR in the overall PCOS vs non-PCOS participants

There was only one study (16) included in our study comparing HOMA-IR between the PCOS and non-PCOS groups. The results showed a significantly higher HOMA-IR in women with PCOS than in those without PCOS. Finally, due to the small number of studies included, we were unable to evaluate publication bias.

## 4 Discussion

We report here an update of previous systematic review and meta-analysis to obtain more information about the correlation of IGFBP-1 levels and PCOS patients with different characteristics, taking account of the contribution of obesity and insulin resistance. In our meta-analysis, we observed a consistent significant decrease in the IGFBP-1 levels in PCOS patients, which was consistent with previous study results (14). Interestingly, IGFBP-1 levels were also consistent significantly decreased in overweight individuals, whether with or without PCOS. This means the feature of decreased IGFBP-1 appears to not be unique to patients with PCOS. Elevated fasting blood insulin is metabolic features in adult women with PCOS, as well as one of the regulators (suppressor) of IGFBP-1 synthesis in the liver. Therefore, we next considered the role of insulin in IGFBP-1 levels. Our results found that in PCOS patients, fasting insulin levels significantly increased, which was consistent with the reduced IGFBP-1 levels in the patients with PCOS. Interestingly, BMI adjustment attenuated associations for fasting insulin and IGFBP-1 levels. Therefore, we speculated that the reduced IGFBP-1 level in PCOS might be related to the increased insulin level, but this correlation can be affected by weight.

Due to the high prevalence of obesity among PCOS patients, there still in controversy whether body weight predominantly influences IGFBP-1 levels in PCOS (9, 25, 26). Thus Kelly et al. had conducted a meta-analysis in 2011, figuring out the decrease of IGFBP-1 in PCOS might be partly attributed to overweight (14). Our meta-analysis updated the data and further analyzed the relationship between insulin and IGFBP-1 in PCOS. We drew similar conclusions with Kelly et al. on the relationship between IGFBP-1 and PCOS. This finding could be explained by the hyperinsulinemia among obesity, which suppresses IGFBP-1 gene transcription in hepatocytes through phosphorylating the Foxo-1 transcription factors (27, 28) and leads to circulating IGFBP-1 reduction (29). However, different from Kelly et al., our results showed a descending trend of IGFBP-1 in PCOS after adjusting for obesity, which may be due to the enlarged sample pool. After further BMI stratification, the results showed that no differences in IGFBP-1 level were observed between the PCOS group and the non-PCOS group in overweight women. However, the difference of IGFBP-1 levels between the PCOS group and the non-PCOS group came close to statistical significance in non-overweight patients. This suggests that weight may have a greater effect on IGFBP-1 level than PCOS itself in overweight women, while PCOS itself may still have an independent effect on IGFBP-1 level in non-overweight women.

Both overweight individuals and women with PCOS have been reported to have increased insulin levels (4). Meanwhile, studies have confirmed that high concentrations of insulin can downregulate the expression of IGFBP-1. In view of this, our meta-analysis compared fasting insulin in PCOS participants classified by BMI. After adjustment of BMI, PCOS itself was independently associated with increased fasting insulin. HOMI-IR also showed a similar trend. This means that PCOS itself is independent factors leading to increased fasting insulin, which is consistent with recent reports that PCOS was independently associated with insulin resistance. IR can synergize with LH to stimulate ovarian androgen production (4, 8, 30, 31). For non-PCOS women, although we observed a trend of higher fasting insulin level in overweight women, no significant differences were observed. We speculate that a decline in IGFBP-1 level may not be completely mediated by fasting insulin level in overweight women. This may, to some extent, provide evidence of whether obesity-related hyperinsulinemia is involved in the regulation of IGFBP-1 (32, 33).

**Figure 5:**
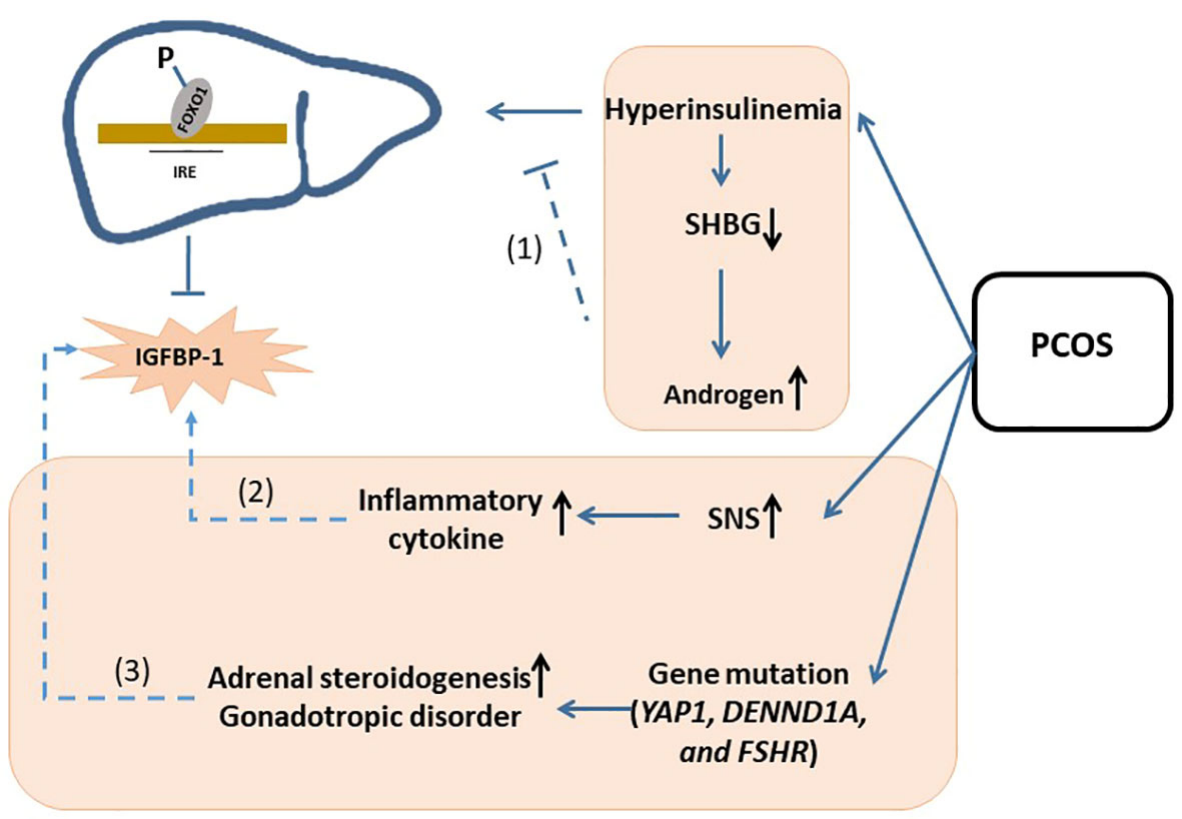
Schematic of hypotheses concerning the mechanisms underlying the change of IGFBP-1 in PCOS. Possible hypothesis was as follow: (1) hyperinsulinemia in PCOS patients may predispose to decreasing the sex hormone-binding globulin (SHBG), which leaves free testosterone increased. So, the higher testosterone level in PCOS may partially consume fasting insulin, which may diminish the suppression of hyperinsulinemia on IGFBP-1 by phosphorylating the Foxo-1 transcription factors; (2) along with hyperandrogenism, the overactivation of central sympathetic nervous system (SNS) may promote inflammatory cytokine production, which can improve the IGFBP-1 level; (3) some gene mutations, including YAP1 overexpression, DENND1A, and FSHR gene mutation, can cause steroidogenic or gonadotropic disorders independently of insulin resistance. IGFBP-1, Insulin-like Growth Factor Binding Protein-1; IGF-1, Insulin-like Growth Factor; PCOS, Polycystic Ovary Syndrome; SHBG, Sex Hormone-binding Globulin; IRE, Insulin-response element; FOX, Forkhead box O; SNS, Sympathetic Nervous System.

Interestingly, after adjusting for BMI, we found that decline in IGFBP-1 in PCOS was not as significantly as the rise in fasting insulin, which was different from obesity patients. We offered the several possible explanations for this phenomenon. First, hyperinsulinemia in PCOS patients may predispose to decreasing the sex hormone-binding globulin (SHBG), which leaves free testosterone increased. So, the higher testosterone level in PCOS may partially consume fasting insulin, which may diminish the suppression of hyperinsulinemia on IGFBP-1 by phosphorylating the Foxo-1 transcription factors (4, 28, 34). Second, along with hyperandrogenism, the overactivation of central sympathetic nervous system (SNS) may promote inflammatory cytokine production (4, 35, 36), which can improve the IGFBP-1 level (33). Third, some gene mutations, including *YAP1* overexpression, *DENND1A*, and *FSHR* gene mutation, can cause steroidogenic or gonadotropic disorders independently of insulin resistance (33, 37–39). IGFBP-1 may not be abnormally decreased in these patients. In conclusion, given its pathological complexity, although IGFBP-1 was statistically declined in PCOS, the diagnosis specificity on IGFBP-1 for PCOS was still needed to be investigated. (Hypotheses were summarized in **Figure 5**).

Heterogeneity

The main source of heterogeneity was different study designs. In the comparison of IGBFP-1 in overall entities (**Figure 2A**), some studies used overweight or obese individuals as the control group, while others selected control candidates with a normal BMI. After excluding researches with the overweight or obese control group, the result remained stable and the heterogeneity dropped. Furthermore, when we compared IGFBP-1 according to the segment of BMIs (**Figures 2B**, **3B**), the study by Firmansyah et al. showed higher IGFBP-1 in overweight PCOS patients, which was opposite to the findings of the other eight studies. The controversy might be explained with the possibilities: Firmansyah, A et al. chose participants with HOMA-IR >2.00, while other studies did not specify HOMA-IR. Thus, insulin resistance in the overweight PCOS group in Firmansyah, A et al. might more serious than other studies, consuming more insulin and leading to a less circulating concentration of insulin, and finally indirectly diminishing the inhibitory effect on hepatic IGFBP-1 production (**Figure 3B**). Meanwhile, hepatic insulin resistance blunts insulin’s inhibition on IGFBP-1 (4), leading to a higher circulating IGFBP-1 in PCOS (**Figure 3B**). The following pooled analyses shown in **Figures 2C**, **3A–C** and **4A–C** included three or fewer studies, which caused the heterogeneity in those meta-analyses. Moreover, there were differences in race, country and cutoff points of BMI in our enrolled articles, which might be potential heterogeneity sources. So, we conducted sensitivity analysis to find the source of heterogeneity, by which the race, country and cutoff points of BMI did not showed strong relativity with heterogeneity.

Innovativeness

Ouranalysis updated the article of C.J. Kelly et al. in 2011, finding out the similar conclusion that reduction of IGFBP-1 was more influenced by comorbid obesity than PCOS itself. However, we found that after BMI stratification, decreased trend on IGFBP-1 in non-overweight PCOS came close to statistical difference, whilst overweight PCOS did show such tendency. This suggested that weight might have a greater effect on IGFBP-1 level than PCOS itself in overweight women, while PCOS itself may still have an independent effect on IGFBP-1 level in non-overweight women. Furthermore, our analysis considered another role of insulin on the change of IGFBP-1. Our work revealed that the decline of IGFBP-1 in PCOS was not as significant as the rise in fasting insulin. This suggested that different from healthy people with declined of IGFBP-1 level as insulin increased, the suppression of PCOS-related hyperinsulinemia on IGFBP-1 seemed weakened under this abnormal circumstance. So, our work provided more clues for the study of potential mechanism in IGFBP-1 influence on PCOS patients.

Limitation

There were some limitations in our study. Firstly, due to a small number of clinical studies, we enrolled some older studies, which might cause some bias in our meta-analysis. Meanwhile, we want to observe the change of IGFBP-1 in two populations: either PCOS versus non-PCOS, or an overweight subgroup versus the normal weight subgroup in either population. So, in view of the two reasons, it was a limitation that meta-analyses did not enroll all 12 studies simultaneously. Secondly, our study only observed the inverse trends of IGFBP-1 and fasting insulin in PCOS, which cannot directly reflect the correlation between IGFBP-1 and insulin in PCOS. Thirdly, because of limited number of studies exploring HOMA-IR, our pooled analysis did not fully estimate insulin resistance for the included population.

## 5 Conclusion

This meta-analysis provides evidence that the decrease of IGFBP-1 in PCOS was more strongly influenced by comorbid obesity than by PCOS itself. Additionally, contrast to previous findings that insulin significantly suppresses IGFBP-1, our results suggested that the suppression of PCOS-related hyperinsulinemia on IGFBP-1 seemed diminished. Overall, our work may provide a novel perspective on the mechanism between insulin and IGFBP-1 underlying PCOS development.

## Data availability statement

The original contributions presented in the study are included in the article/**Supplementary Material**. Further inquiries can be directed to the corresponding authors.

## Author contributions

BG: Conceptualization, Supervision, Writing – review & editing. YJ: Conceptualization, Data curation, Formal analysis, Investigation, Methodology, Project administration, Software, Writing – original draft, Writing – review & editing. FS: Data curation, Methodology, Writing – review & editing. AY: Methodology, Writing – original draft. XY: Writing – review & editing. YL: Methodology, Writing – original draft. SL: Formal analysis, Writing – original draft. XJ: Data curation, Writing – original draft. KW: Conceptualization, Project administration, Writing – original draft. LZ: Formal analysis, Writing – original draft. SX: Formal analysis, Writing – original draft. WZ: Data curation, Formal analysis, Writing – original draft. XW: Data curation, Formal analysis, Writing – original draft. GZ: Conceptualization, Supervision, Writing – review & editing.
