# Supplementary material for: Insulin-like growth factor binding protein-1 and insulin in polycystic ovary syndrome: a systematic review and meta-analysis
Source: Front Endocrinol (Lausanne). 2023 Dec 15;14:1279717. doi: 10.3389/fendo.2023.1279717 (PMC10762309; doi:10.3389/fendo.2023.1279717)
Supplement: Supplementary file 1 [file DataSheet_1.pdf]

Table S1 Quality assessment according to *JBI Critical Appraisal Checklist for analytical cross-sectional study*

Assessment Items	1 Were the criteria for inclusion in the sample clearly defined?	2 Were the study subjects and the setting described in detail?	3 Was the exposure measured in a valid and reliable way?	4 Were objective, standard criteria used for measurement of the condition?	5 Were confounding factors identified?	6 Were strategies to deal with confounding factors stated?	7 Were the outcomes measured in a valid and reliable way?	8 Was appropriate statistical analysis used?
2018Firmansyah, A et al.	Yes	Yes	Yes	Yes	Yes	Yes	Yes	Yes
2003Silfen et al.	Yes	Yes	Yes	Yes	Unclear	No	Yes	Yes
2002Garcia-Rudaz et al.	Yes	Yes	Yes	Yes	Unclear	No	Yes	Yes
2001Kowalska, I et al.	Yes	Yes	Yes	Yes	Yes	Yes	Yes	Yes
1997Carmina, E et al.	Yes	Yes	Yes	Yes	Yes	Yes	Yes	Yes
1996Morales, A J et al.	Yes	Yes	Yes	Yes	Yes	Yes	Yes	Yes
1996Morris, D V et al.	Yes	Yes	Yes	Yes	Yes	Yes	Yes	Yes
1995Buyalos et al.	Yes	Yes	Yes	Yes	Yes	Yes	Yes	Yes

1993Insler et al.	Yes	Yes	Yes	Yes	Yes	Yes	Yes	Yes
1993Tiitinen, A E et al.	Yes	Yes	Yes	Yes	Yes	Yes	Yes	Yes
1990Laatikainen et al.	Yes	Yes	Yes	Yes	Yes	Yes	Yes	Yes
1990Iwashita M et al.	Yes	Yes	Yes	Yes	Yes	Yes	Yes	Yes
Overall appraisal	Included yes							

Figure S1 Sensitivity analysis of IGFBP-1 in PCOS versus control group

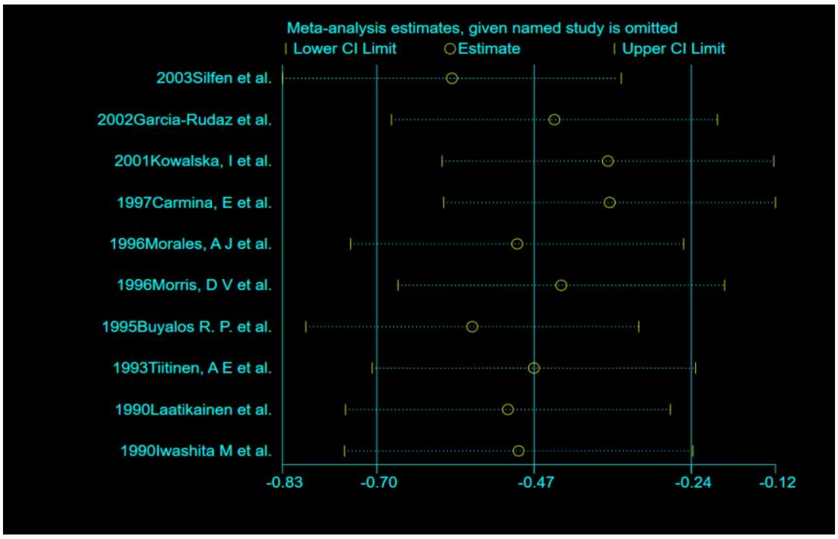

Figrure S2 Subgroup analysis of IGFBP-1 in PCOS versus control group

Study or Subgroup	PCOS Mean	SD	Total	Control Mean	SD	Total	Weight	Std. Mean Difference IV, Random, 95% CI	Year
1990Laatikainen et al.	54.53	41.23	20	50.69	13.62	6	10.3%	0.10 [-0.81, 1.01]	1990
1990Iwashita M et al.	32.3	27.53	26	43.66	33.36	30	25.0%	-0.36 [-0.89, 0.17]	1990
1993Tiitinen, A E et al.	32.05	26.07	23	44	24.6	5	9.1%	-0.45 [-1.42, 0.53]	1993
1995Buyalos et al.	3.92	4.49	16	2.98	1.58	16	16.3%	0.27 [-0.42, 0.97]	1995
1996Morris, D V et al.	31.43	28.71	15	56	18.66	10	11.7%	-0.94 [-1.79, -0.09]	1996
1996Morales, A J et al.	2.07	2.84	16	2.78	2.54	16	16.3%	-0.26 [-0.95, 0.44]	1996
1997Carmina, E et al.	1.86	2.44	25	6	3.49	15	0.0%	-1.41 [-2.13, -0.69]	1997
2001Kowalska, I et al.	10.74	7.83	42	34.1	33.5	11	0.0%	-1.40 [-2.12, -0.68]	2001
2002Garcia-Rudaz, M C et al.	9.9	11.9	13	24	20.88	10	11.3%	-0.83 [-1.70, 0.04]	2002
2003Silfen, M E et al.	1.53	2.26	33	1	0.8	15	0.0%	0.27 [-0.34, 0.88]	2003
**Total (95% CI)**			**129**			**93**	**100.0%**	**-0.32 [-0.63, -0.01]**	

Heterogeneity: $Tau^2 = 0.03$; $Chi^2 = 7.10$, df = 6 (P = 0.31); $I^2 = 16\%$
Test for overall effect: Z = 2.04 (P = 0.04)

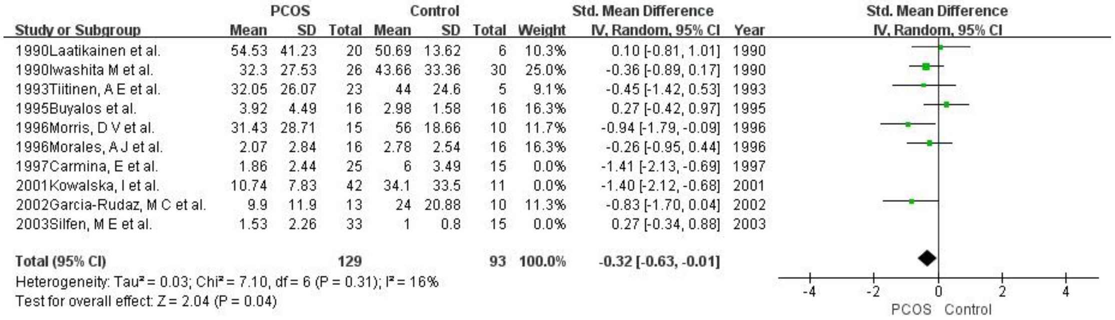

Figure S3 Begg's test and Egger's test for publication bias assessment on IGFBP-1 in PCOS versus control group

```
Tests for Publication Bias

Begg's Test

  adj. Kendall's Score (P-Q) =       -5
          Std. Dev. of Score =    11.18
           Number of Studies =       10
                           z =    -0.45
                    Pr > |z| =    0.655
                           z =     0.36 (continuity corrected)
                    Pr > |z| =    0.721 (continuity corrected)

Egger's test
```

Std_Eff	Coef.	Std. Err.	t	P>|t|	[95% Conf. Interval]	
slope	.3013732	1.179268	0.26	0.805	-2.418023	3.02077
bias	-2.122453	3.203203	-0.66	0.526	-9.509053	5.264147

Figure S4 Funnel plot of IGFBP-1 in PCOS versus control

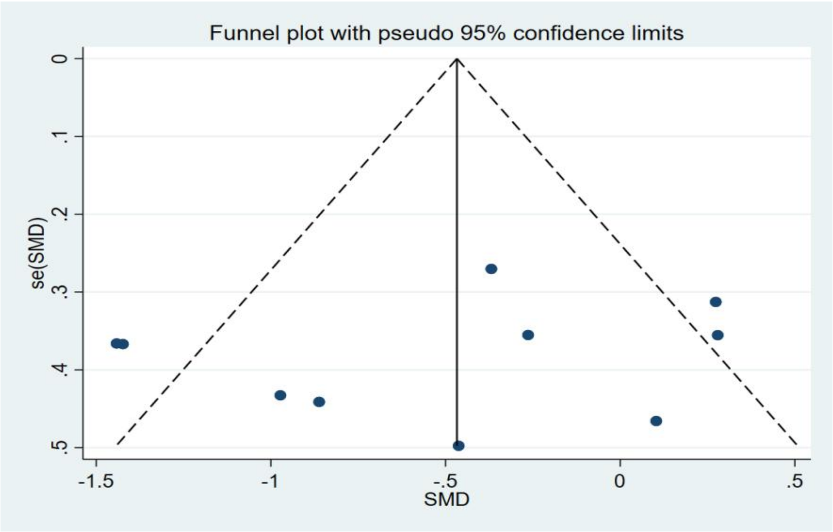

Figure S5 Sensitivity analysis of IGFBP-1 in PCOS with or without overweight

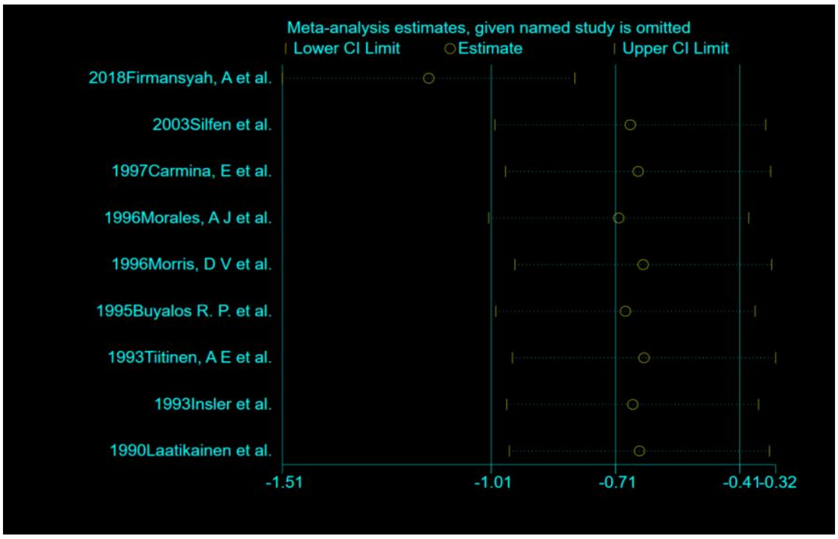

Figrure S6 Subgroup analysis of IGFBP-1 in PCOS with or without overweight

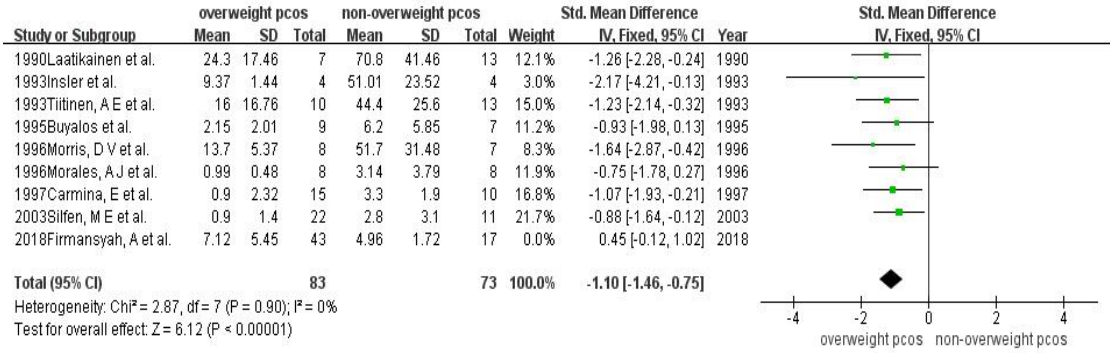

Figure S7 Begg's test and Egger's test for publication bias assessment on IGFBP-1 between overweight and non-overweight PCOS

```
Tests for Publication Bias

Begg's Test

  adj. Kendall's Score (P-Q) =     -18
          Std. Dev. of Score =    9.59
           Number of Studies =       9
                          z  =   -1.88
                    Pr > |z| =   0.061
                          z  =    1.77 (continuity corrected)
                    Pr > |z| =   0.076 (continuity corrected)

Egger's test
```

Std_Eff	Coef.	Std. Err.	t	P>|t|	[95% Conf.	Interval]
slope	1.476677	.526408	2.81	0.026	.2319197	2.721434
bias	-4.991912	1.149069	-4.34	0.003	-7.709029	-2.274796

Figure S8 Funnel plot of IGFBP-1 between overweight and non-overweight PCOS

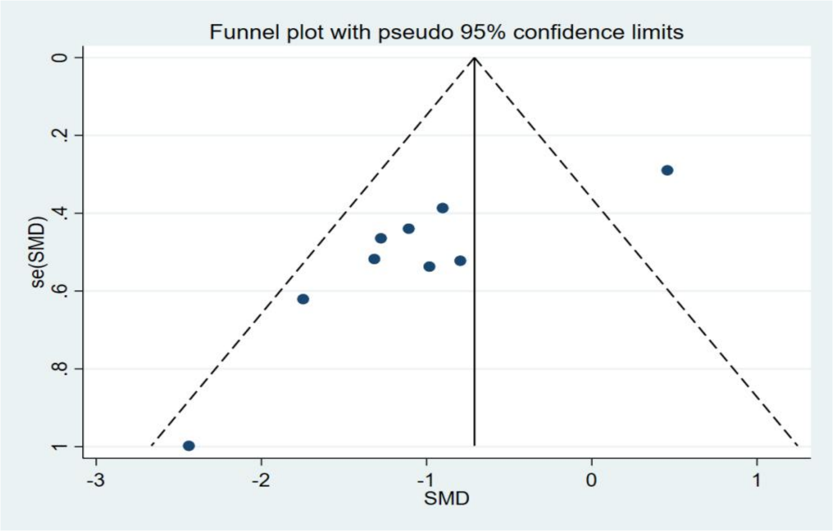

Figure S9 Begg's Test and Egger's test for publication bias assessment on IGFBP-1 between overweight and non-overweight PCOS after eliminating 2018Firmansyah, A et al.

```
Tests for Publication Bias

Begg's Test

  adj. Kendall's Score (P-Q) =     -16
          Std. Dev. of Score =    8.08
           Number of Studies =       8
                          z  =   -1.98
                    Pr > |z| =   0.048
                          z  =    1.86 (continuity corrected)
                    Pr > |z| =   0.063 (continuity corrected)

Egger's test
```

Std_Eff	Coef.	Std. Err.	t	P>|t|	[95% Conf.	Interval]
slope	.0322548	.386278	0.08	0.936	-.9129333	.977443
bias	-2.407023	.7599203	-3.17	0.019	-4.266481	-.5475649

Figure S10 Funnel plot of IGFBP-1 between overweight and non-overweight PCOS after eliminating 2018Firmansyah, A et al..

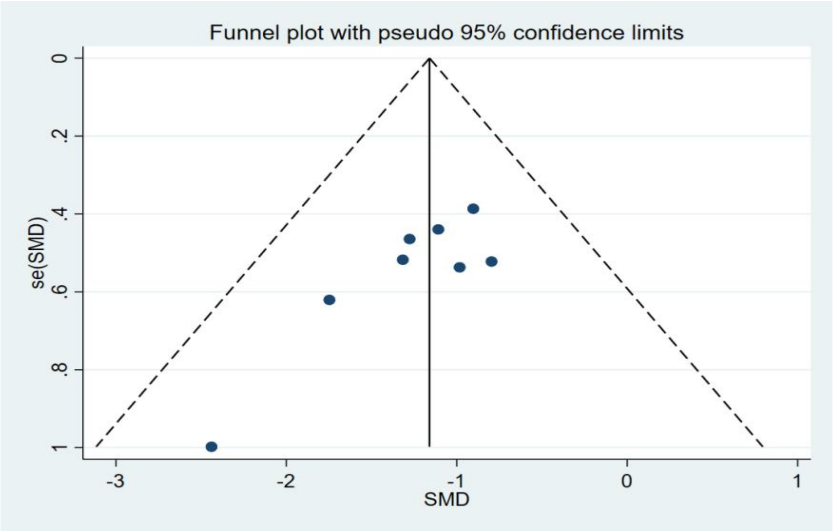

Figure S11 Begg's test and Egger's test for publication bias assessment on IGFBP-1 in control group with or without overweight

```
Tests for Publication Bias

Begg's Test

  adj. Kendall's Score (P-Q) =        6
          Std. Dev. of Score =     2.94
           Number of Studies =        4
                          z  =     2.04
                    Pr > |z| =    0.042
                          z  =     1.70 (continuity corrected)
                    Pr > |z| =    0.089 (continuity corrected)
```

Egger's test

Std_Eff	Coef.	Std. Err.	t	P>|t|	[95% Conf.	Interval]
slope	-6.445071	.4987455	-12.92	0.006	-8.591	-4.299143
bias	9.116719	.9073289	10.05	0.010	5.212797	13.02064

Figure S12 Trim-and-fill method to correct publication bias on IGFBP-1 in control group with or without overweight

```
Meta-analysis

       |  Pooled      95% CI         Asymptotic      No. of
Method |     Est   Lower   Upper  z_value  p_value   studies
-------+----------------------------------------------------
Fixed  |  -1.463  -2.001  -0.924   -5.322    0.000       4
Random |  -1.435  -2.053  -0.817   -4.548    0.000

Test for heterogeneity: Q=  3.906 on 3 degrees of freedom (p= 0.272)
Moment-based estimate of between studies variance =  0.093

Trimming estimator: Linear
Meta-analysis type: Fixed-effects model

iteration |  estimate    Tn    # to trim     diff
----------+--------------------------------------
    1     |   -1.463      7         1          10
    2     |   -1.641      7         1           0

Filled
Meta-analysis

       |  Pooled      95% CI         Asymptotic      No. of
Method |     Est   Lower   Upper  z_value  p_value   studies
-------+----------------------------------------------------
Fixed  |  -1.641  -2.137  -1.145   -6.489    0.000       5
Random |  -1.622  -2.267  -0.977   -4.928    0.000

Test for heterogeneity: Q=  6.649 on 4 degrees of freedom (p= 0.156)
Moment-based estimate of between studies variance =  0.215
```

Figure S13 Sensitivity analysis of IGFBP-1 in overweight participants with or without PCOS

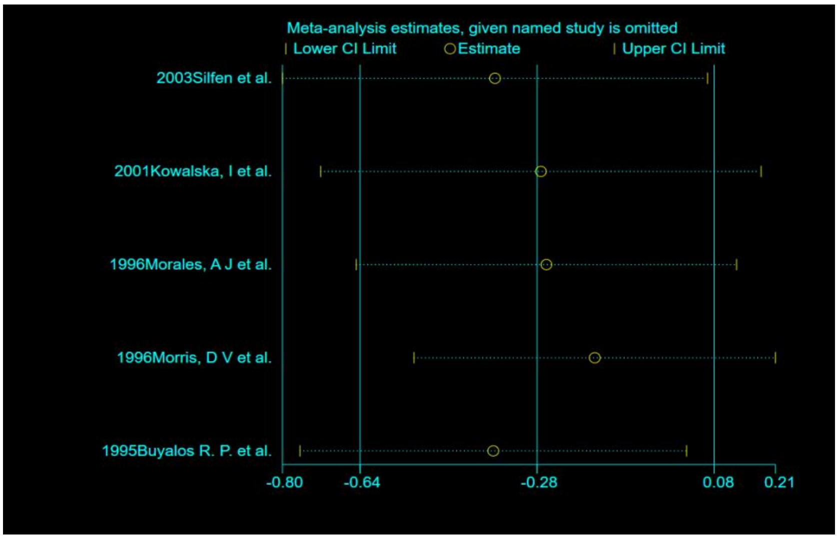

Figure S14 Subgroup analysis of IGFBP-1 in overweight participants with or without PCOS

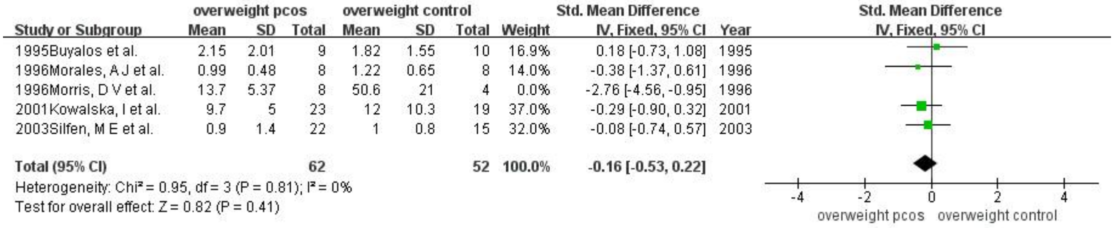

Study or Subgroup	overweight pcos Mean	SD	Total	overweight control Mean	SD	Total	Weight	Std. Mean Difference IV, Fixed, 95% CI	Year
1995Buyalos et al.	2.15	2.01	9	1.82	1.55	10	16.9%	0.18 [-0.73, 1.08]	1995
1996Morales, A J et al.	0.99	0.48	8	1.22	0.65	8	14.0%	-0.38 [-1.37, 0.61]	1996
1996Morris, D V et al.	13.7	5.37	8	50.6	21	4	0.0%	-2.76 [-4.56, -0.95]	1996
2001Kowalska, I et al.	9.7	5	23	12	10.3	19	37.0%	-0.29 [-0.90, 0.32]	2001
2003Silfen, M E et al.	0.9	1.4	22	1	0.8	15	32.0%	-0.08 [-0.74, 0.57]	2003
**Total (95% CI)**			**62**			**52**	**100.0%**	**-0.16 [-0.53, 0.22]**	

Heterogeneity: Chi² = 0.95, df = 3 (P = 0.81); I² = 0%
Test for overall effect: Z = 0.82 (P = 0.41)

Figure S15 Begg's test and Egger's test for publication bias assessment on IGFBP-1 in overweight

participants with or without PCOS

```
Tests for Publication Bias

Begg's Test

  adj. Kendall's Score (P-Q) =       -4
          Std. Dev. of Score =     4.08
           Number of Studies =        5
                          z  =    -0.98
                Pr > |z| =    0.327
                          z  =     0.73 (continuity corrected)
                Pr > |z| =    0.462 (continuity corrected)
```

Egger's test

Std_Eff	Coef.	Std. Err.	t	P>|t|	[95% Conf.	Interval]
slope	1.079413	.7286586	1.48	0.235	-1.239504	3.39833
bias	-3.446908	1.756105	-1.96	0.144	-9.035618	2.141802

Figure S16 Funnel plot of IGFBP-1 in overweight participants with or without PCOS

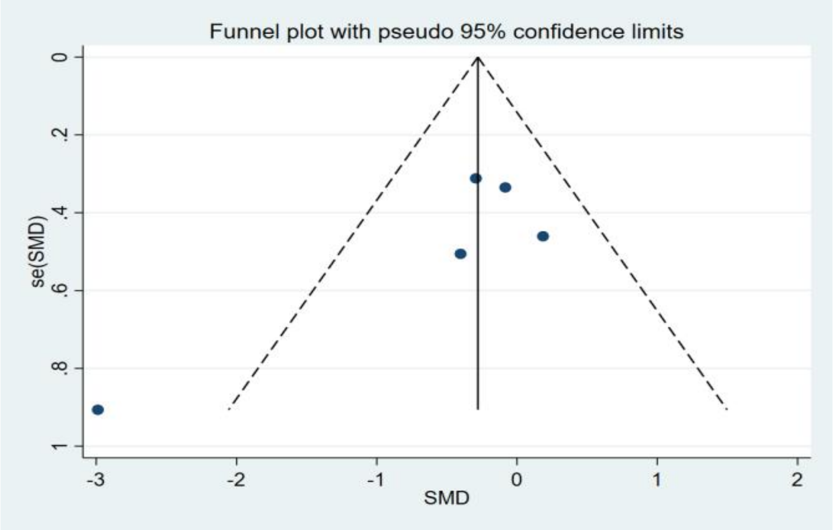

Figure S17 Begg's test and Egger's test for publication bias assessment on IGFBP-1 in nonoverweight participants with or without PCOS

```
Tests for Publication Bias

Begg's Test

  adj. Kendall's Score (P-Q) =       0
          Std. Dev. of Score =    8.08
           Number of Studies =       8
                          z  =    0.00
                    Pr > |z| =   1.000
                          z  =   -0.12 (continuity corrected)
                    Pr > |z| =   1.000 (continuity corrected)
```

Egger's test

Std_Eff	Coef.	Std. Err.	t	P>|t|	[95% Conf. Interval]	
slope	-.6180164	.6641092	-0.93	0.388	-2.243033	1.007
bias	.7645243	1.516771	0.50	0.632	-2.946882	4.47593

Figure S18 Funnel plot of IGFBP-1 in nonoverweight participants with or without PCOS

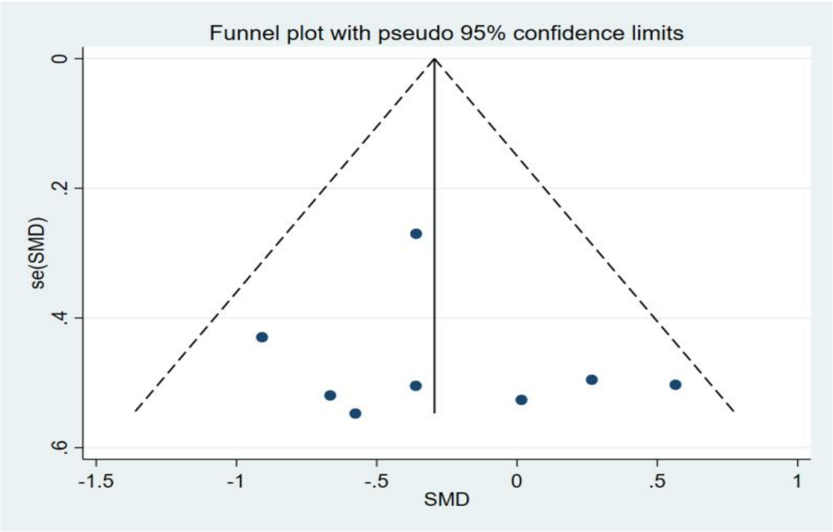

Figure S19 Begg's test and Egger's test for publication bias assessment on fasting insulin in PCOS patients versus the control population

```
Tests for Publication Bias

Begg's Test

  adj. Kendall's Score (P-Q) =      -4
          Std. Dev. of Score =    8.08
           Number of Studies =       8
                          z  =   -0.49
                    Pr > |z| =   0.621
                          z  =    0.37 (continuity corrected)
                    Pr > |z| =   0.711 (continuity corrected)
```

Egger's test

Std_Eff	Coef.	Std. Err.	t	P>|t|	[95% Conf. Interval]	
slope	.3237934	.7737522	0.42	0.690	-1.56951	2.217097
bias	.8632054	2.066983	0.42	0.691	-4.19452	5.920931

Figure S20 Sensitivity analysis of fasting inuslin in PCOS with or without overweight

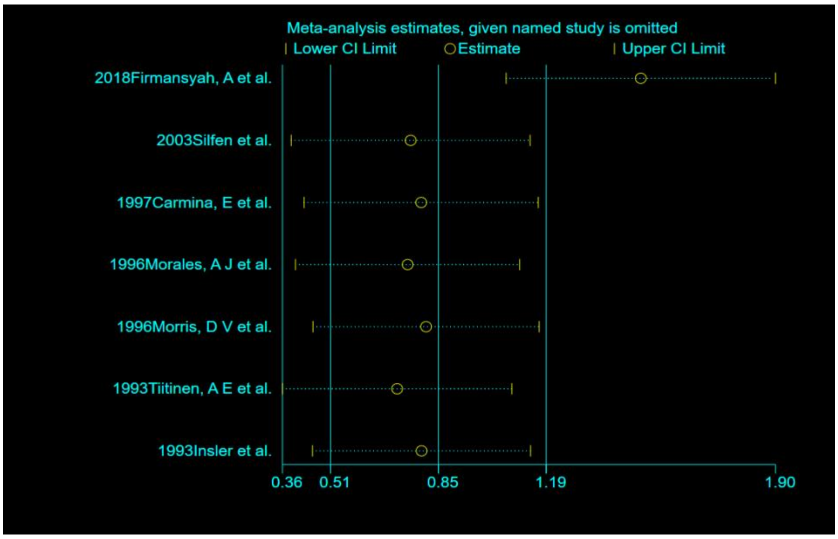

Figure S21 Subgroup analysis of fasting insulin in PCOS with or without overweight

Study or Subgroup	overweight pcos Mean	SD	Total	non-overweight pcos Mean	SD	Total	Weight	Std. Mean Difference IV, Fixed, 95% CI	Year
1993Tiitinen, A E et al.	15.9	6.96	10	5.6	4.33	13	18.1%	1.77 [0.77, 2.77]	1993
1993Insler et al.	15.68	2.64	4	8	2.22	4	3.3%	2.74 [0.40, 5.08]	1993
1996Morris, D V et al.	26.8	15.56	8	12.6	2.38	7	14.2%	1.16 [0.04, 2.28]	1996
1996Morales, A J et al.	50.11	28.12	8	7.2	9.36	8	11.5%	1.94 [0.69, 3.18]	1996
1997Carmina, E et al.	16.44	6.93	15	9.3	4.93	10	23.9%	1.11 [0.24, 1.98]	1997
2003Silfen, M E et al.	30.7	18.3	22	12	3.6	11	29.0%	1.20 [0.41, 1.99]	2003
2018Firmansyah, A et al.	13.58	7.351	43	15.44	3.623	17	0.0%	-0.28 [-0.84, 0.28]	2018
**Total (95% CI)**			**67**			**53**	**100.0%**	**1.41 [0.99, 1.83]**	

Heterogeneity: Chi² = 3.35, df = 5 (P = 0.65); I² = 0%
Test for overall effect: Z = 6.52 (P < 0.00001)

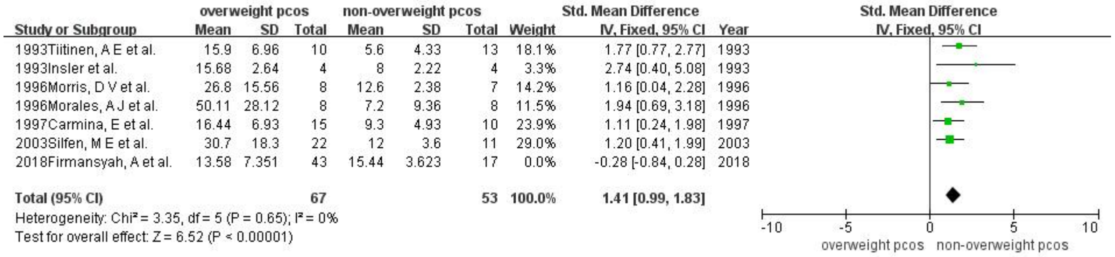

Figure S22 Begg's test and Egger's test for publication bias assessment on fasting insulin in PCOS with or without overweight

```
Tests for Publication Bias

Begg's Test

  adj. Kendall's Score (P-Q) =       9
          Std. Dev. of Score =    6.66
           Number of Studies =       7
                           z  =    1.35
                    Pr > |z|  =   0.176
                           z  =    1.20 (continuity corrected)
                    Pr > |z|  =   0.230 (continuity corrected)

Egger's test
```

Std_Eff	Coef.	Std. Err.	t	P>|t|	[95% Conf.	Interval]
slope	-1.329708	.603737	-2.20	0.079	-2.881664	.2222472
bias	5.082802	1.32334	3.84	0.012	1.681047	8.484557

Figure S23 Begg's test and Egger's test for publication bias assessment on fasting insulin in overweight participants with or without PCOS

```
Tests for Publication Bias

Begg's Test

  adj. Kendall's Score (P-Q) =       4
          Std. Dev. of Score =    2.94
           Number of Studies =       4
                          z  =    1.36
                    Pr > |z| =   0.174
                          z  =    1.02 (continuity corrected)
                    Pr > |z| =   0.308 (continuity corrected)

Egger's test
```

Std_Eff	Coef.	Std. Err.	t	P>|t|	[95% Conf.	Interval]
slope	-.9032433	.6611387	-1.37	0.305	-3.747894	1.941407
bias	3.479741	1.388909	2.51	0.129	-2.496253	9.455735

Figure S24 Begg's test and Egger's test for publication bias assessment on fasting insulin in non-overweight participants with or without PCOS

```
Tests for Publication Bias

Begg's Test

  adj. Kendall's Score (P-Q) =      -4
          Std. Dev. of Score =    4.08
           Number of Studies =       5
                          z  =   -0.98
                    Pr > |z| =   0.327
                          z  =    0.73 (continuity corrected)
                    Pr > |z| =   0.462 (continuity corrected)

Egger's test
```

Std_Eff	Coef.	Std. Err.	t	P>|t|	[95% Conf.	Interval]
slope	1.129133	.7707967	1.46	0.239	-1.323886	3.582152
bias	-1.638469	1.870797	-0.88	0.446	-7.592181	4.315243
