# Supplementary material for: Insulin-like growth factor binding protein-1 and insulin in polycystic ovary syndrome: a systematic review and meta-analysis
Source: Front Endocrinol (Lausanne). 2023 Dec 15;14:1279717. doi: 10.3389/fendo.2023.1279717 (PMC10762309; doi:10.3389/fendo.2023.1279717)
Supplement: Supplementary file 2 [file DataSheet_2.pdf]

Search strategy in Pubmed

#1 "Insulin-Like Growth Factor Binding Protein 1"[Mesh]

#2 (((Insulin Like Growth Factor Binding Protein 1[Title/Abstract]) OR (IGFBP-1[Title/Abstract])) OR (IGF-Binding Protein 1[Title/Abstract])) OR (IGF Binding Protein 1[Title/Abstract])

#3 #1 OR #2

#4 ((("Polycystic Ovary Syndrome"[Mesh]) OR (Stein Leventhal Syndrome[Title/Abstract])) OR (Ovarian Degeneration, Sclerocystic[Title/Abstract])) OR (Sclerocystic Ovaries[Title/Abstract])

#5 #3 AND #4

Search strategy in EMBASE

#1 'somatomedin binding protein 1'/exp

#2 'insulin like growth factor binding protein 1':ab,ti OR 'insulin-like growth factor binding protein 1':ab,ti OR 'insulin-like growth-factor binding protein 1':ab,ti

#3 #1 OR #2

#4 'ovary polycystic disease'/exp

#5 'cystic ovary':ab,ti OR 'micropolycystic ovary':ab,ti OR 'multiple follicle cyst':ab,ti OR 'ovary polycystic syndrome':ab,ti OR 'ovary micropolycystic':ab,ti OR 'polycystic ovarian disease':ab,ti OR 'polycystic ovary':ab,ti OR 'polycystic ovary syndrome':ab,ti OR 'stein cohen leventhal syndrome':ab,ti OR 'stein leventhal':ab,ti

#6 #4 OR #5

#7 #3 AND #6

Search strategy associated in the Cochrane Library

"Insulin-Like Growth Factor Binding Protein 1" OR "IGFBP-1" OR "IGF-Binding Protein 1" OR "IGF Binding Protein 1":ti,ab,kw AND "Polycystic Ovary Syndrome" OR "Stein Leventhal Syndrome" OR "Ovarian Degeneration, Sclerocystic" OR "Sclerocystic Ovaries":ti,ab,kw (Word variations have been searched)

Search strategy in Web of Scienc

#1 TS=(Insulin-Like Growth Factor Binding Protein 1 or Insulin Like Growth Factor Binding Protein 1 or IGFBP-1 or IGF-Binding Protein 1 or IGF Binding Protein 1)

#2 TS=(Polycystic Ovary Syndrome or Stein Leventhal Syndrome or Ovarian Degeneration, Sclerocystic or Sclerocystic Ovaries)

#3 #1 AND #2

Search strategy in Ovid MEDLINE

#1 (Insulin-Like Growth Factor Binding Protein 1 or Insulin Like Growth Factor Binding Protein 1 or IGFBP-1 or IGF-Binding Protein 1 or IGF Binding Protein 1).ti,ab,kw

#2 (Polycystic Ovary Syndrome or Stein Leventhal Syndrome or Ovarian Degeneration, Sclerocystic or Sclerocystic Ovaries).ti,ab,kw

#3 #1 AND #2

Search strategy in Scopus

#1 TITLE-ABS-KEY (Insulin-Like Growth Factor Binding Protein 1 or Insulin Like Growth Factor Binding Protein 1 or IGFBP-1 or IGF-Binding Protein 1 or IGF Binding Protein 1)

#2 TITLE-ABS-KEY (Polycystic Ovary Syndrome or Stein Leventhal Syndrome or Ovarian Degeneration, Sclerocystic or Sclerocystic Ovaries)

#3 #1 AND #2
